# On the Treatment of Pyorrhœa Alveolaris

**Published:** 1886-10

**Authors:** J. Henry Whatford

**Affiliations:** Eastbourne.


ARTICLE V.

### ON THE TREATMENT OF PYORRHŒA ALVEOLARIS.

BY J. HENRY WHATFORD, EASTBOURNE.

(Read before Southern Counties' Branch of the British Dental Association.)

The writer refers mainly to a mode of treatment proposed by Dr. Philip Crampton, of Dublin, who was himself a sufferer from pyorrhœa.

He found sulphate of copper act as a complete cure in his own case, and he cured many others similarly affected. For the last two and a half years the writer employed this method of using sulphate of copper in some well marked cases of pyorrhœa alveolaris (two of them very unpromising) and in the great majority found it curative.

Pyorrhœa alveolaris in its early stage appears to be a form of local inflammation resulting in separation of the margin of the gum from the necks of the teeth, and the formation of a pocket for the lodgment of foreign bodies, the gum at the same time changing its color from the normal pale rose hue to a darker color, becoming spongy in texture, swollen, readily bleeding on slight provocation, and its margin becoming rounder and averted from the necks of the teeth. This may exist for months or years, with so little inconvenience as scarcely, if at all, to attract the attention of the patient. As the disease advances the symptoms increase, and there supervenes a sense of fulness, throbbing, and gnawing irritation. Slight pressure on the gum surrounding the teeth causes a drop or more of thick, sometimes fetid pus to exude at the necks of the teeth. The teeth lose their proper sensibility, gradually become loose, and the denuded fangs become often coated with a tenacious layer of very hard greenish-brown tartar, and if the disease be allowed to progress without treatment, the alveoli get absorbed, and the teeth drop out. This disease is found apparently in every constitution, healthy or otherwise; indeed, the general state of constitution seems not to affect the disease. It appears to be sometimes hereditary; local injury seems an exciting cause in some cases.

The local effects of pyorrhœa resemble those of scurvy, however, the last named disease has very marked constitutional symptoms of what pyorrhœa has none.

Scurvy can be generally traced to constitutional degradation, owing to want of proper nutriment. Again, scurvy is generally readily curable by proper nutriment combined with constitutional treatment and cessation from hardship; local treatment being unneeded; whereas in pyorrhœa local treatment is all-important, and constitutional of slight avail. Some refer the origin of pyorrhœa to the alveolo-dental membrane, others believe that the alveolar process is first affected, while others attribute it wholly to neglect in cleaning the teeth, and thus allowing tartar to accumulate between the necks of the teeth and the gum. Mr. Charles Tomes has related a case of a patient, aged 25 years, in which all remaining teeth were removed and on many of them distinctly affected there was no tartar. Dr. Arkovy, of Buda-Pesth, found that, besides pus corpuscles, threads of leptothrix buccalis were always present in abundance, and concluded the fungus was intimately connected with the origin of pyorrhœa alveolaris. Dr. Islai and others have confirmed these results. A case is quoted exemplifying that the purely surgical treatment advocated by Dr. Riggs is not always successful.

Mrs. P., aged 34, no family, somewhat anæmic in appearance, with good family history, and in very good health.

About three years ago she noticed that her upper and lower central incisors were becoming loose, and her dentist prescribed some remedy, which for a time eased her discomfort; the gums, however, became rapidly spongy, the teeth looser, causing considerable discomfort and at times pain. Her gums were scarified; tannin, carbolic acid, acetic acid, and other remedies were tried without avail, and she gave up all idea of further treatment. Coming under Mr. Whatford's care, he decided to try sulphate of copper. At the time of her visit the incisors of the upper and lower jaws, and the bicuspid teeth of the upper jaw were very loose, and the neighboring gum deeply congested, tumid and thickened, and detached from the fangs, which were coated with hard tartar, more particularly those of the lower teeth, and on pressure a thick discharge appeared between the teeth and the gums. For the first two or three days a little finely-powdered sulphate of copper was packed between the gums and teeth by means of a piece of wood suitably shaped, and an improvement in comfort and in the appearance of the gums appeared on the second day. When the gums had improved in color, and the sponginess had disappeared, he removed the tartar, which could not be done earlier without causing pain. For seven successive days he packed the finely-powdered sulphate of copper under the gum and around the teeth in the manner described, and then, after a week's interval, renewed the treatment for four more days. The discharge ceased after the seventh visit, but the upper teeth were soonest well; eight daily applications sufficed to cure them; the lower teeth gave more trouble, probably owing to the difficulty experienced in thoroughly applying the remedy on aceount of the ready flow of saliva it appeared to cause. Eventually, however, all the teeth became quite firm. More than a year since the commencement of treatment the mouth was found to be in perfect health. Two or three applications of sulphate of copper cause such a contraction of spongy gum as to steady the teeth, and make the operation of removing the tartar comparatively easy, and Mr. Whatford removes it at one, or at the outside two visits.

When cases have been of long standing, it is necessary to apply the sulphate of copper at once for eight or ten successive days to ensure immunity from relapse; there is no risk in careful hands in freely using sulphate of copper; it is better even to overdo it a little to make sure of a good result. In early or slight cases fewer applications will suffice. The caustic should be used as a saturated solution, or in powder. Some little pain results from the use of caustic, and this is greatly relieved by holding cold water in the mouth. It is well to prepare the patient for this irritation, which arises at intervals during treatment, lessening gradually, eventually disappearing. The copper taste can be readily removed by a sip or two of bi-carbonate of soda solution, and almost prevented provided the sip be taken while the powder is lying on the gum, and before it becomes mixed up in the mouth by an attempt to expectorate before the sip is taken. In applying sulphate of copper to the gum of teeth in the lower jaw, the saliva ejector may be used; a teaspoonful of a solution of bicarbonate of soda is introduced into the mouth as the bulb of the ejector is withdrawn, the patient being told to take a mouthful of water and shake it well about before expectorating.

The properties of sulphate of copper are then considered. It is as a styptic for bleeding surfaces, a stimulant for ulcers, and an escharotic for warts. It is used as a lotion and as an injection to diminish excessive secretions from mucous membranes; ten or fifteen grains in two ounces of water forms a prompt emetic; half an ounce taken into the stomach would probably kill, so that the quantity employed in the treatment of pyorrhœa could hardly produce toxic symptoms, especially if carbonate of soda in solution be employed as is recommended above.

Mr. Whatford prefers sulphate of copper as the remedy to other caustics, because: (1.) Its action apparently involves less loss of tissues, while its curative power seems to equal any. (2.) It does not blacken the teeth as nitrate of silver does, nor act on their structure as acids do, nor spread over more surface than that intended, as chloride of zinc, caustic potash, and some others are liable to do. (3.) Its application, as a rule, causes little pain; its action as a caustic is so limited that the gum freely granulates under its use, and therefore it can, in most cases be applied daily till cure is apparent without checking these granulations as other caustics are liable to do. (4.) Its continued use leads to no detriment, and in most cases a feeling of relief and comfort arises shortly after its application. —*British Journal of Dental Science.*

